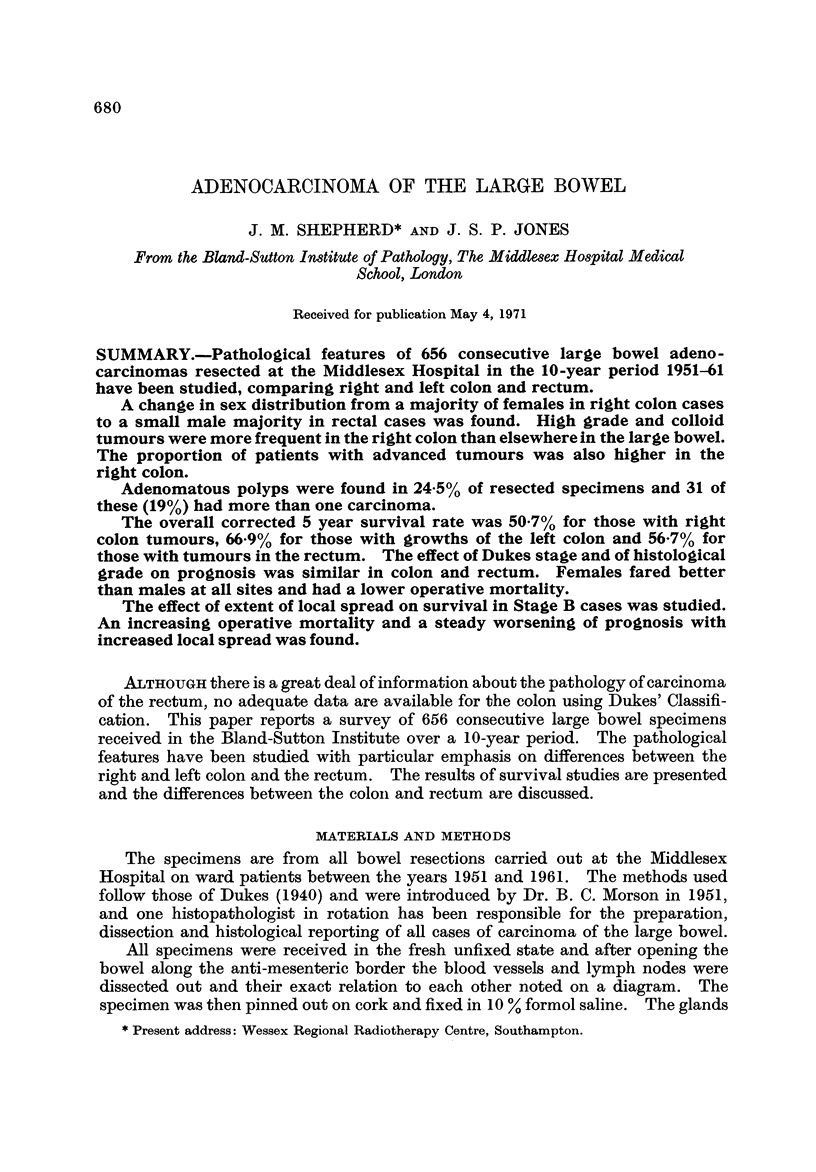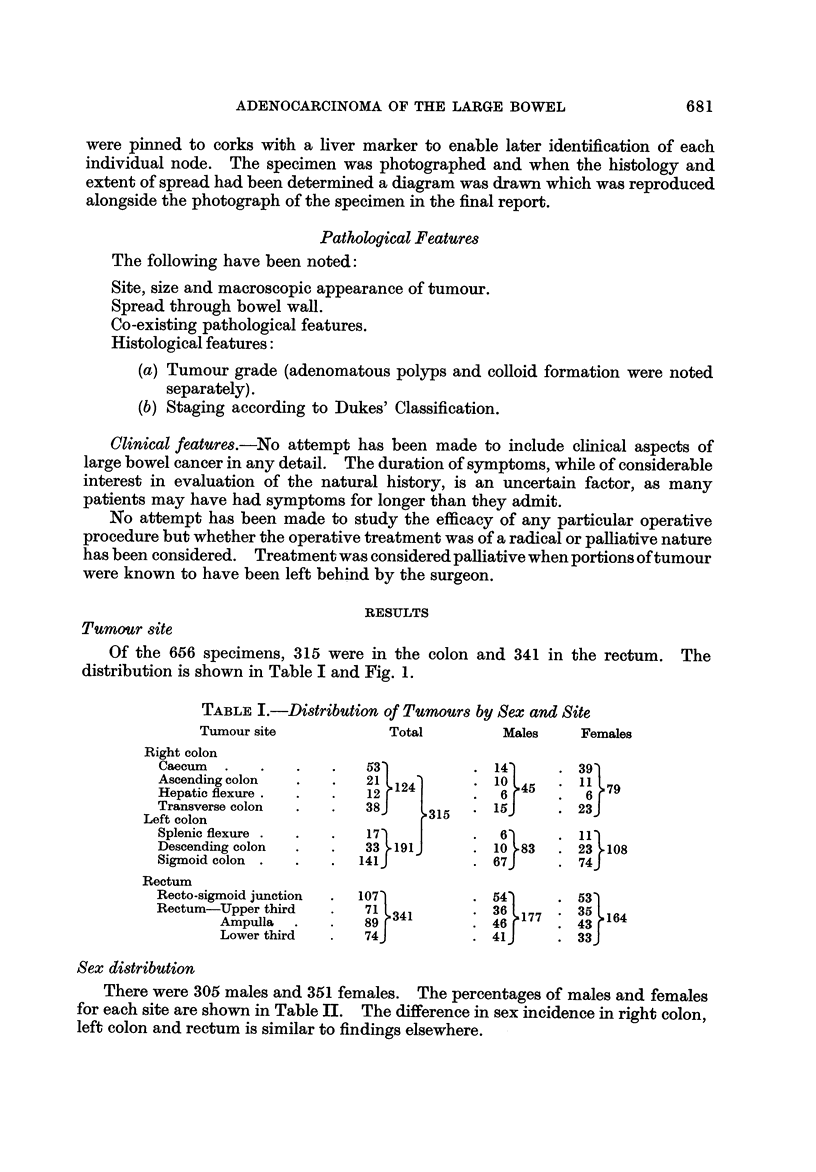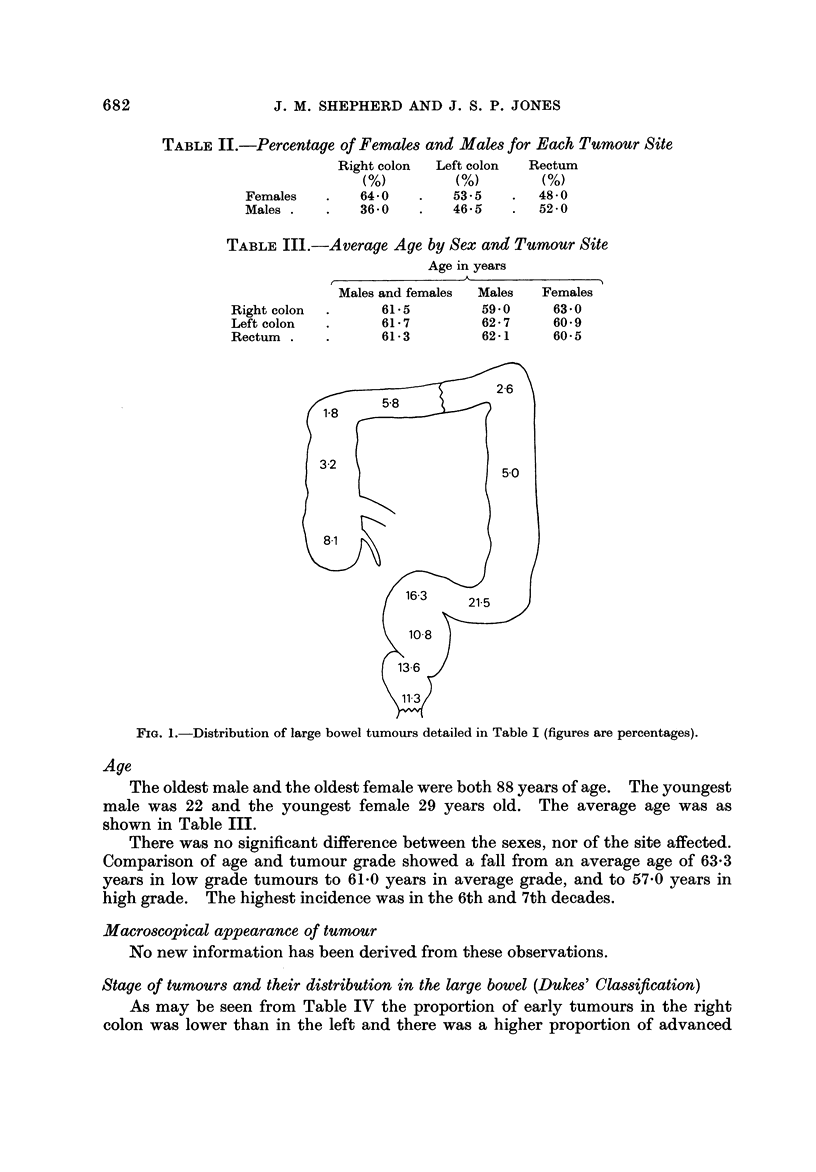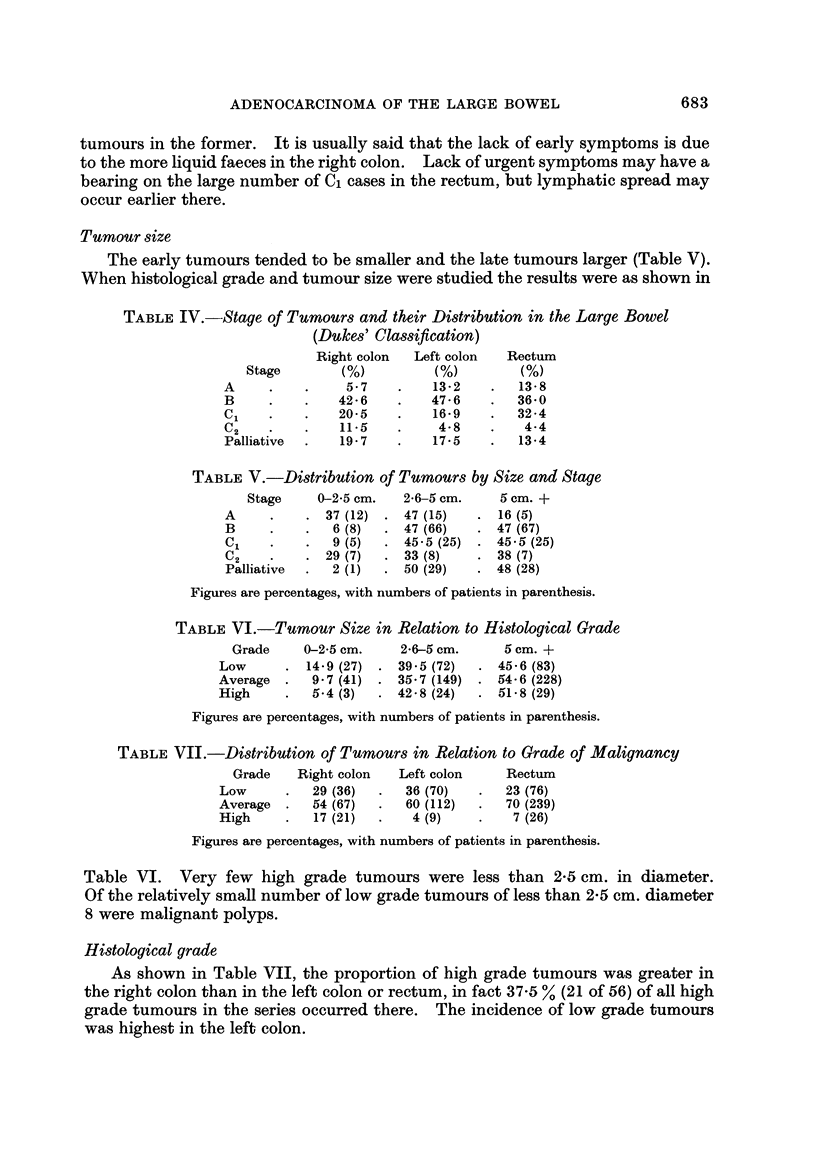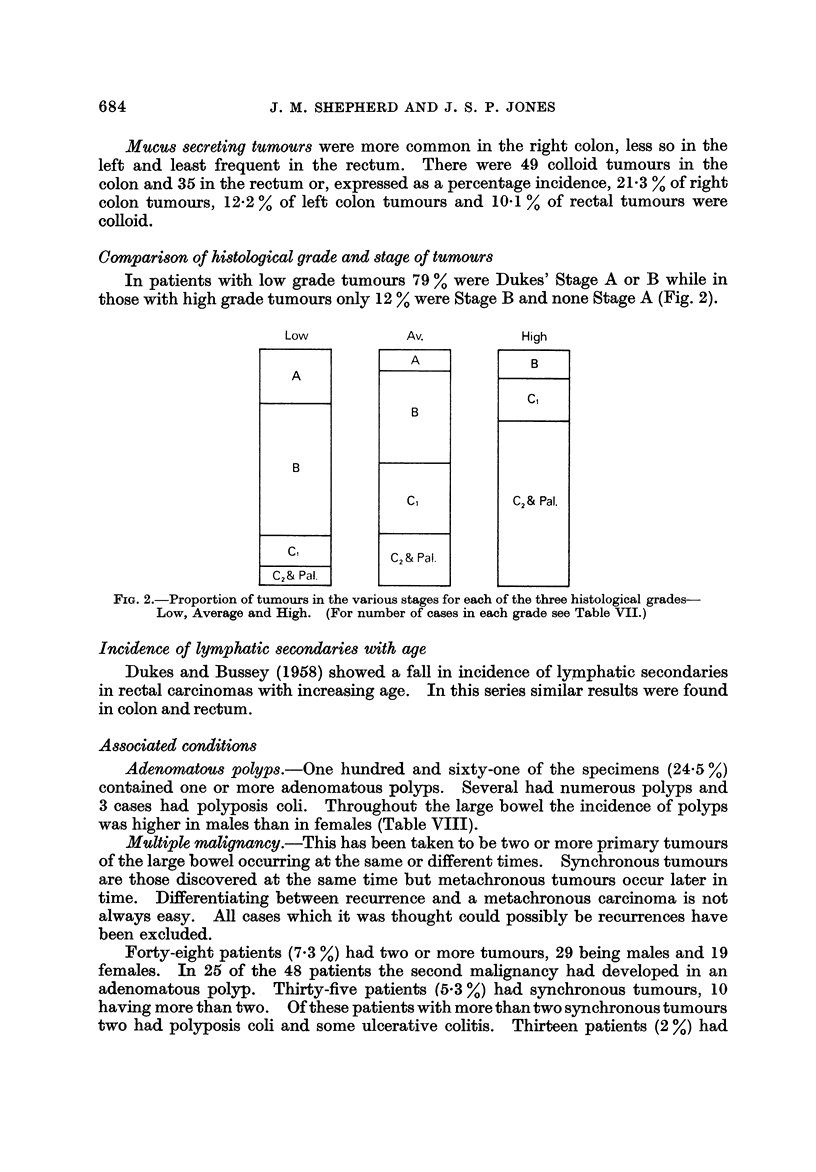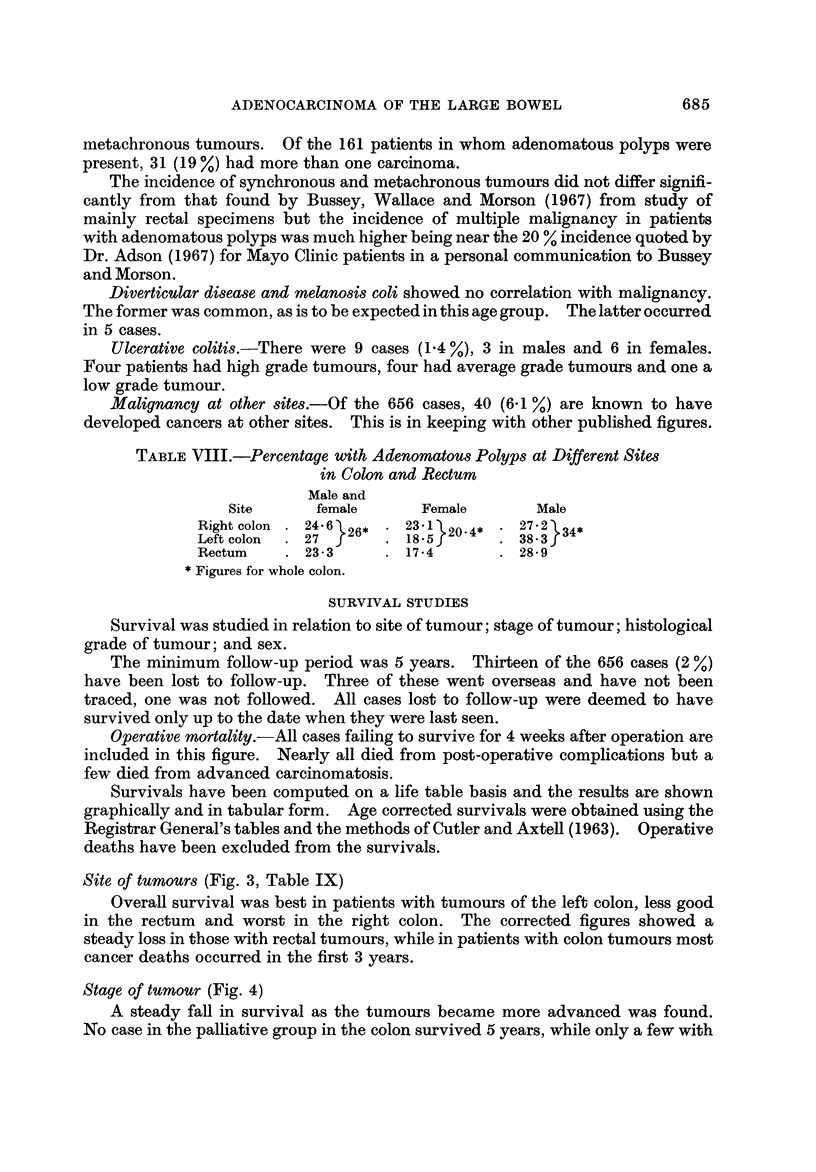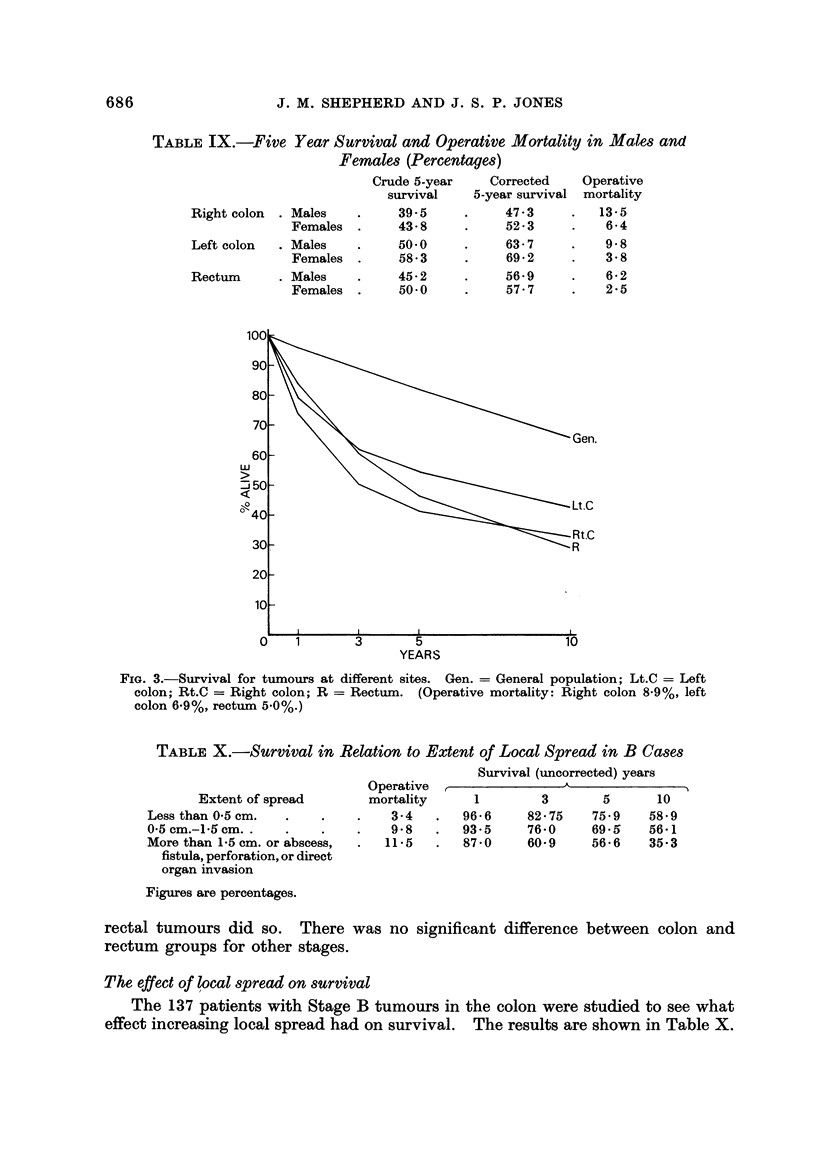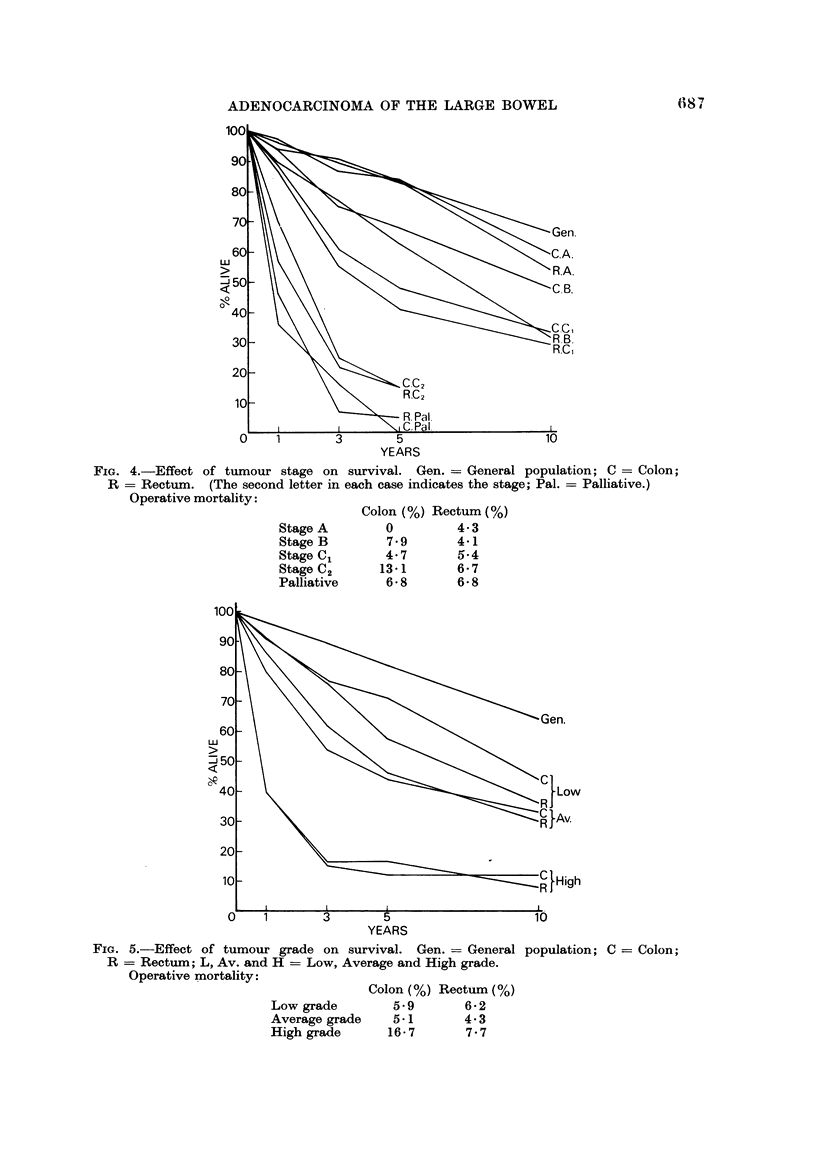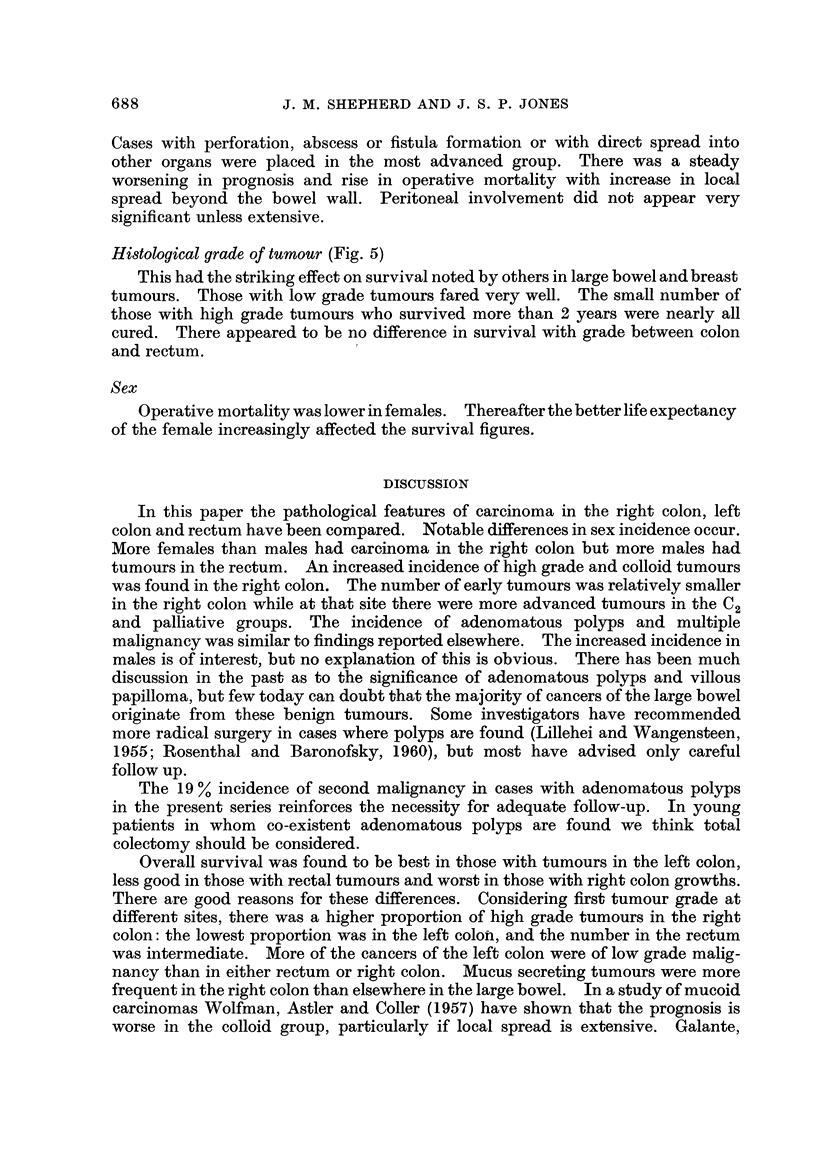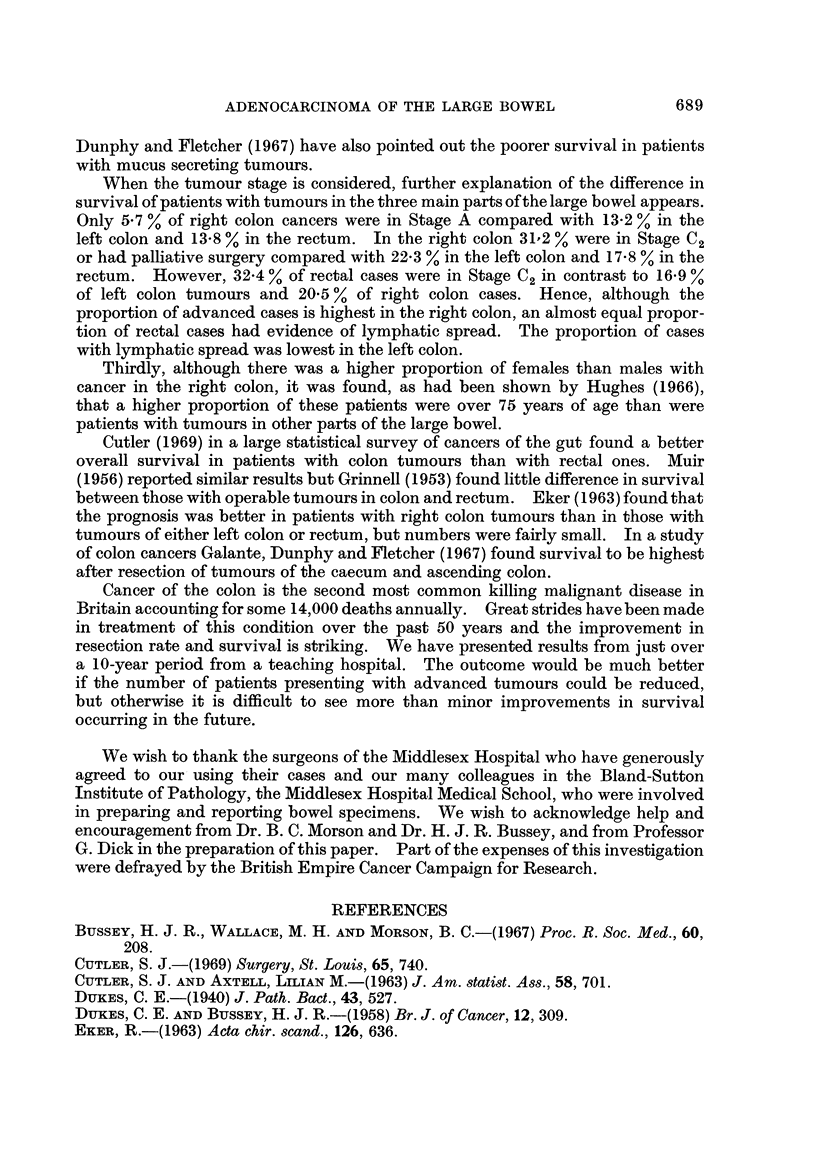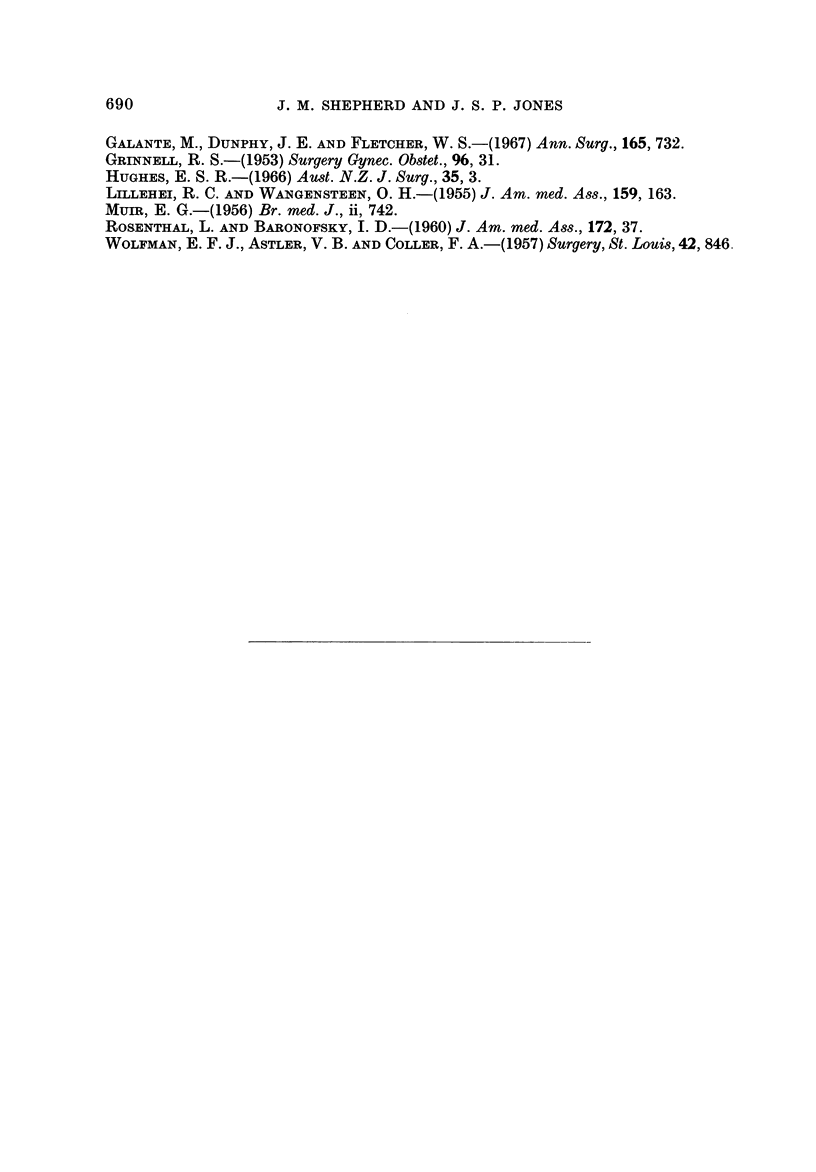# Adenocarcinoma of the Large Bowel

**DOI:** 10.1038/bjc.1971.83

**Published:** 1971-12

**Authors:** J. M. Shepherd, J. S. P. Jones

## Abstract

Pathological features of 656 consecutive large bowel adenocarcinomas resected at the Middlesex Hospital in the 10-year period 1951-61 have been studied, comparing right and left colon and rectum.

A change in sex distribution from a majority of females in right colon cases to a small male majority in rectal cases was found. High grade and colloid tumours were more frequent in the right colon than elsewhere in the large bowel. The proportion of patients with advanced tumours was also higher in the right colon.

Adenomatous polyps were found in 24·5% of resected specimens and 31 of these (19%) had more than one carcinoma.

The overall corrected 5 year survival rate was 50·7% for those with right colon tumours, 66·9% for those with growths of the left colon and 56·7% for those with tumours in the rectum. The effect of Dukes stage and of histological grade on prognosis was similar in colon and rectum. Females fared better than males at all sites and had a lower operative mortality.

The effect of extent of local spread on survival in Stage B cases was studied. An increasing operative mortality and a steady worsening of prognosis with increased local spread was found.


### ADENOCARCINOMA OF THE LARGE BOWEL

J. M. SHEPHERD* AND J. S. P. JONES

*From the Bland-Sutton Institute of Pathology, The Middlesex Hospital Medical School, London*

Received for publication May 4, 1971

**SUMMARY.—Pathological features of 656 consecutive large bowel adenocarcinomas resected at the Middlesex Hospital in the 10-year period 1951–61 have been studied, comparing right and left colon and rectum.**

**A change in sex distribution from a majority of females in right colon cases to a small male majority in rectal cases was found. High grade and colloid tumours were more frequent in the right colon than elsewhere in the large bowel. The proportion of patients with advanced tumours was also higher in the right colon.**

**Adenomatous polyps were found in 24·5% of resected specimens and 31 of these (19%) had more than one carcinoma.**

**The overall corrected 5 year survival rate was 50·7% for those with right colon tumours, 66·9% for those with growths of the left colon and 56·7% for those with tumours in the rectum. The effect of Dukes stage and of histological grade on prognosis was similar in colon and rectum. Females fared better than males at all sites and had a lower operative mortality.**

**The effect of extent of local spread on survival in Stage B cases was studied. An increasing operative mortality and a steady worsening of prognosis with increased local spread was found.**

ALTHOUGH there is a great deal of information about the pathology of carcinoma of the rectum, no adequate data are available for the colon using Dukes' Classification. This paper reports a survey of 656 consecutive large bowel specimens received in the Bland-Sutton Institute over a 10-year period. The pathological features have been studied with particular emphasis on differences between the right and left colon and the rectum. The results of survival studies are presented and the differences between the colon and rectum are discussed.

### MATERIALS AND METHODS

The specimens are from all bowel resections carried out at the Middlesex Hospital on ward patients between the years 1951 and 1961. The methods used follow those of Dukes (1940) and were introduced by Dr. B. C. Morson in 1951, and one histopathologist in rotation has been responsible for the preparation, dissection and histological reporting of all cases of carcinoma of the large bowel.

All specimens were received in the fresh unfixed state and after opening the bowel along the anti-mesenteric border the blood vessels and lymph nodes were dissected out and their exact relation to each other noted on a diagram. The specimen was then pinned out on cork and fixed in 10 % formol saline. The glands

* Present address: Wessex Regional Radiotherapy Centre, Southampton.

were pinned to corks with a liver marker to enable later identification of each individual node. The specimen was photographed and when the histology and extent of spread had been determined a diagram was drawn which was reproduced alongside the photograph of the specimen in the final report.

### *Pathological Features*

The following have been noted:

Site, size and macroscopic appearance of tumour.
Spread through bowel wall.
Co-existing pathological features.
Histological features:

(*a*) Tumour grade (adenomatous polyps and colloid formation were noted separately).
(*b*) Staging according to Dukes' Classification.

*Clinical features.*—No attempt has been made to include clinical aspects of large bowel cancer in any detail. The duration of symptoms, while of considerable interest in evaluation of the natural history, is an uncertain factor, as many patients may have had symptoms for longer than they admit.

No attempt has been made to study the efficacy of any particular operative procedure but whether the operative treatment was of a radical or palliative nature has been considered. Treatment was considered palliative when portions of tumour were known to have been left behind by the surgeon.

### RESULTS

### *Tumour site*

Of the 656 specimens, 315 were in the colon and 341 in the rectum. The distribution is shown in Table I and Fig. 1.

TABLE I.—*Distribution of Tumours by Sex and Site*

| Tumour site | Total | | | Males | | Females | |
|---|---|---|---|---|---|---|---|
| Right colon | | | | | | | |
| Caecum | 53 | | | 14 | | 39 | |
| Ascending colon | 21 | | | 10 | | 11 | |
| Hepatic flexure | 12 | 124 | | 6 | 45 | 6 | 79 |
| Transverse colon | 38 | | | 15 | | 23 | |
| Left colon | | | 315 | | | | |
| Splenic flexure | 17 | | | 6 | | 11 | |
| Descending colon | 33 | 191 | | 10 | 83 | 23 | 108 |
| Sigmoid colon | 141 | | | 67 | | 74 | |
| Rectum | | | | | | | |
| Recto-sigmoid junction | 107 | | | 54 | | 53 | |
| Rectum—Upper third | 71 | | | 36 | | 35 | |
| Ampulla | 89 | 341 | | 46 | 177 | 43 | 164 |
| Lower third | 74 | | | 41 | | 33 | |

### *Sex distribution*

There were 305 males and 351 females. The percentages of males and females for each site are shown in Table II. The difference in sex incidence in right colon, left colon and rectum is similar to findings elsewhere.

TABLE II.—*Percentage of Females and Males for Each Tumour Site*

| | Right colon (%) | Left colon (%) | Rectum (%) |
|---|---|---|---|
| Females | 64·0 | 53·5 | 48·0 |
| Males | 36·0 | 46·5 | 52·0 |

TABLE III.—*Average Age by Sex and Tumour Site*

| | Age in years | | |
|---|---|---|---|
| | Males and females | Males | Females |
| Right colon | 61·5 | 59·0 | 63·0 |
| Left colon | 61·7 | 62·7 | 60·9 |
| Rectum | 61·3 | 62·1 | 60·5 |

FIG. 1.—Distribution of large bowel tumours detailed in Table I (figures are percentages).

*Age*

The oldest male and the oldest female were both 88 years of age. The youngest male was 22 and the youngest female 29 years old. The average age was as shown in Table III.

There was no significant difference between the sexes, nor of the site affected. Comparison of age and tumour grade showed a fall from an average age of 63·3 years in low grade tumours to 61·0 years in average grade, and to 57·0 years in high grade. The highest incidence was in the 6th and 7th decades.

*Macroscopical appearance of tumour*

No new information has been derived from these observations.

*Stage of tumours and their distribution in the large bowel (Dukes' Classification)*

As may be seen from Table IV the proportion of early tumours in the right colon was lower than in the left and there was a higher proportion of advanced tumours in the former. It is usually said that the lack of early symptoms is due to the more liquid faeces in the right colon. Lack of urgent symptoms may have a bearing on the large number of $C_1$ cases in the rectum, but lymphatic spread may occur earlier there.

*Tumour size*

The early tumours tended to be smaller and the late tumours larger (Table V). When histological grade and tumour size were studied the results were as shown in

TABLE IV.—*Stage of Tumours and their Distribution in the Large Bowel (Dukes' Classification)*

| Stage | Right colon (%) | Left colon (%) | Rectum (%) |
|---|---|---|---|
| A | 5·7 | 13·2 | 13·8 |
| B | 42·6 | 47·6 | 36·0 |
| $C_1$ | 20·5 | 16·9 | 32·4 |
| $C_2$ | 11·5 | 4·8 | 4·4 |
| Palliative | 19·7 | 17·5 | 13·4 |

TABLE V.—*Distribution of Tumours by Size and Stage*

| Stage | 0–2·5 cm. | 2·6–5 cm. | 5 cm. + |
|---|---|---|---|
| A | 37 (12) | 47 (15) | 16 (5) |
| B | 6 (8) | 47 (66) | 47 (67) |
| $C_1$ | 9 (5) | 45·5 (25) | 45·5 (25) |
| $C_2$ | 29 (7) | 33 (8) | 38 (7) |
| Palliative | 2 (1) | 50 (29) | 48 (28) |

Figures are percentages, with numbers of patients in parenthesis.

TABLE VI.—*Tumour Size in Relation to Histological Grade*

| Grade | 0–2·5 cm. | 2·6–5 cm. | 5 cm. + |
|---|---|---|---|
| Low | 14·9 (27) | 39·5 (72) | 45·6 (83) |
| Average | 9·7 (41) | 35·7 (149) | 54·6 (228) |
| High | 5·4 (3) | 42·8 (24) | 51·8 (29) |

Figures are percentages, with numbers of patients in parenthesis.

TABLE VII.—*Distribution of Tumours in Relation to Grade of Malignancy*

| Grade | Right colon | Left colon | Rectum |
|---|---|---|---|
| Low | 29 (36) | 36 (70) | 23 (76) |
| Average | 54 (67) | 60 (112) | 70 (239) |
| High | 17 (21) | 4 (9) | 7 (26) |

Figures are percentages, with numbers of patients in parenthesis.

Table VI. Very few high grade tumours were less than 2·5 cm. in diameter. Of the relatively small number of low grade tumours of less than 2·5 cm. diameter 8 were malignant polyps.

*Histological grade*

As shown in Table VII, the proportion of high grade tumours was greater in the right colon than in the left colon or rectum, in fact 37·5 % (21 of 56) of all high grade tumours in the series occurred there. The incidence of low grade tumours was highest in the left colon.

*Mucus secreting tumours* were more common in the right colon, less so in the left and least frequent in the rectum. There were 49 colloid tumours in the colon and 35 in the rectum or, expressed as a percentage incidence, 21·3 % of right colon tumours, 12·2 % of left colon tumours and 10·1 % of rectal tumours were colloid.

*Comparison of histological grade and stage of tumours*

In patients with low grade tumours 79 % were Dukes' Stage A or B while in those with high grade tumours only 12 % were Stage B and none Stage A (Fig. 2).

FIG. 2.—Proportion of tumours in the various stages for each of the three histological grades—Low, Average and High. (For number of cases in each grade see Table VII.)

*Incidence of lymphatic secondaries with age*

Dukes and Bussey (1958) showed a fall in incidence of lymphatic secondaries in rectal carcinomas with increasing age. In this series similar results were found in colon and rectum.

*Associated conditions*

*Adenomatous polyps.*—One hundred and sixty-one of the specimens (24·5 %) contained one or more adenomatous polyps. Several had numerous polyps and 3 cases had polyposis coli. Throughout the large bowel the incidence of polyps was higher in males than in females (Table VIII).

*Multiple malignancy.*—This has been taken to be two or more primary tumours of the large bowel occurring at the same or different times. Synchronous tumours are those discovered at the same time but metachronous tumours occur later in time. Differentiating between recurrence and a metachronous carcinoma is not always easy. All cases which it was thought could possibly be recurrences have been excluded.

Forty-eight patients (7·3 %) had two or more tumours, 29 being males and 19 females. In 25 of the 48 patients the second malignancy had developed in an adenomatous polyp. Thirty-five patients (5·3 %) had synchronous tumours, 10 having more than two. Of these patients with more than two synchronous tumours two had polyposis coli and some ulcerative colitis. Thirteen patients (2 %) had metachronous tumours. Of the 161 patients in whom adenomatous polyps were present, 31 (19 %) had more than one carcinoma.

The incidence of synchronous and metachronous tumours did not differ significantly from that found by Bussey, Wallace and Morson (1967) from study of mainly rectal specimens but the incidence of multiple malignancy in patients with adenomatous polyps was much higher being near the 20 % incidence quoted by Dr. Adson (1967) for Mayo Clinic patients in a personal communication to Bussey and Morson.

*Diverticular disease and melanosis coli* showed no correlation with malignancy. The former was common, as is to be expected in this age group. The latter occurred in 5 cases.

*Ulcerative colitis.*—There were 9 cases (1·4 %), 3 in males and 6 in females. Four patients had high grade tumours, four had average grade tumours and one a low grade tumour.

*Malignancy at other sites.*—Of the 656 cases, 40 (6·1 %) are known to have developed cancers at other sites. This is in keeping with other published figures.

TABLE VIII.—*Percentage with Adenomatous Polyps at Different Sites in Colon and Rectum*

| Site | Male and female | | Female | | Male | |
|---|---|---|---|---|---|---|
| Right colon | 24·6 | 26* | 23·1 | 20·4* | 27·2 | 34* |
| Left colon | 27 | | 18·5 | | 38·3 | |
| Rectum | 23·3 | | 17·4 | | 28·9 | |

* Figures for whole colon.

### SURVIVAL STUDIES

Survival was studied in relation to site of tumour; stage of tumour; histological grade of tumour; and sex.

The minimum follow-up period was 5 years. Thirteen of the 656 cases (2 %) have been lost to follow-up. Three of these went overseas and have not been traced, one was not followed. All cases lost to follow-up were deemed to have survived only up to the date when they were last seen.

*Operative mortality.*—All cases failing to survive for 4 weeks after operation are included in this figure. Nearly all died from post-operative complications but a few died from advanced carcinomatosis.

Survivals have been computed on a life table basis and the results are shown graphically and in tabular form. Age corrected survivals were obtained using the Registrar General's tables and the methods of Cutler and Axtell (1963). Operative deaths have been excluded from the survivals.

### *Site of tumours* (Fig. 3, Table IX)

Overall survival was best in patients with tumours of the left colon, less good in the rectum and worst in the right colon. The corrected figures showed a steady loss in those with rectal tumours, while in patients with colon tumours most cancer deaths occurred in the first 3 years.

### *Stage of tumour* (Fig. 4)

A steady fall in survival as the tumours became more advanced was found. No case in the palliative group in the colon survived 5 years, while only a few with

TABLE IX.—*Five Year Survival and Operative Mortality in Males and Females (Percentages)*

| | | Crude 5-year survival | Corrected 5-year survival | Operative mortality |
|---|---|---|---|---|
| Right colon | Males | 39·5 | 47·3 | 13·5 |
| | Females | 43·8 | 52·3 | 6·4 |
| Left colon | Males | 50·0 | 63·7 | 9·8 |
| | Females | 58·3 | 69·2 | 3·8 |
| Rectum | Males | 45·2 | 56·9 | 6·2 |
| | Females | 50·0 | 57·7 | 2·5 |

FIG. 3.—Survival for tumours at different sites. Gen. = General population; Lt.C = Left colon; Rt.C = Right colon; R = Rectum. (Operative mortality: Right colon 8·9%, left colon 6·9%, rectum 5·0%.)

TABLE X.—*Survival in Relation to Extent of Local Spread in B Cases*

| Extent of spread | Operative mortality | Survival (uncorrected) years 1 | 3 | 5 | 10 |
|---|---|---|---|---|---|
| Less than 0·5 cm. | 3·4 | 96·6 | 82·75 | 75·9 | 58·9 |
| 0·5 cm.–1·5 cm. | 9·8 | 93·5 | 76·0 | 69·5 | 56·1 |
| More than 1·5 cm. or abscess, fistula, perforation, or direct organ invasion | 11·5 | 87·0 | 60·9 | 56·6 | 35·3 |

Figures are percentages.

rectal tumours did so. There was no significant difference between colon and rectum groups for other stages.

*The effect of local spread on survival*

The 137 patients with Stage B tumours in the colon were studied to see what effect increasing local spread had on survival. The results are shown in Table X.

Fig. 4.—Effect of tumour stage on survival. Gen. = General population; C = Colon; R = Rectum. (The second letter in each case indicates the stage; Pal. = Palliative.)

Operative mortality:

| | Colon (%) | Rectum (%) |
|---|---|---|
| Stage A | 0 | 4·3 |
| Stage B | 7·9 | 4·1 |
| Stage $C_1$ | 4·7 | 5·4 |
| Stage $C_2$ | 13·1 | 6·7 |
| Palliative | 6·8 | 6·8 |

Fig. 5.—Effect of tumour grade on survival. Gen. = General population; C = Colon; R = Rectum; L, Av. and H = Low, Average and High grade.

Operative mortality:

| | Colon (%) | Rectum (%) |
|---|---|---|
| Low grade | 5·9 | 6·2 |
| Average grade | 5·1 | 4·3 |
| High grade | 16·7 | 7·7 |

Cases with perforation, abscess or fistula formation or with direct spread into other organs were placed in the most advanced group. There was a steady worsening in prognosis and rise in operative mortality with increase in local spread beyond the bowel wall. Peritoneal involvement did not appear very significant unless extensive.

*Histological grade of tumour* (Fig. 5)

This had the striking effect on survival noted by others in large bowel and breast tumours. Those with low grade tumours fared very well. The small number of those with high grade tumours who survived more than 2 years were nearly all cured. There appeared to be no difference in survival with grade between colon and rectum.

*Sex*

Operative mortality was lower in females. Thereafter the better life expectancy of the female increasingly affected the survival figures.

### DISCUSSION

In this paper the pathological features of carcinoma in the right colon, left colon and rectum have been compared. Notable differences in sex incidence occur. More females than males had carcinoma in the right colon but more males had tumours in the rectum. An increased incidence of high grade and colloid tumours was found in the right colon. The number of early tumours was relatively smaller in the right colon while at that site there were more advanced tumours in the $C_2$ and palliative groups. The incidence of adenomatous polyps and multiple malignancy was similar to findings reported elsewhere. The increased incidence in males is of interest, but no explanation of this is obvious. There has been much discussion in the past as to the significance of adenomatous polyps and villous papilloma, but few today can doubt that the majority of cancers of the large bowel originate from these benign tumours. Some investigators have recommended more radical surgery in cases where polyps are found (Lillehei and Wangensteen, 1955; Rosenthal and Baronofsky, 1960), but most have advised only careful follow up.

The 19% incidence of second malignancy in cases with adenomatous polyps in the present series reinforces the necessity for adequate follow-up. In young patients in whom co-existent adenomatous polyps are found we think total colectomy should be considered.

Overall survival was found to be best in those with tumours in the left colon, less good in those with rectal tumours and worst in those with right colon growths. There are good reasons for these differences. Considering first tumour grade at different sites, there was a higher proportion of high grade tumours in the right colon: the lowest proportion was in the left colon, and the number in the rectum was intermediate. More of the cancers of the left colon were of low grade malignancy than in either rectum or right colon. Mucus secreting tumours were more frequent in the right colon than elsewhere in the large bowel. In a study of mucoid carcinomas Wolfman, Astler and Coller (1957) have shown that the prognosis is worse in the colloid group, particularly if local spread is extensive. Galante,

Dunphy and Fletcher (1967) have also pointed out the poorer survival in patients with mucus secreting tumours.

When the tumour stage is considered, further explanation of the difference in survival of patients with tumours in the three main parts of the large bowel appears. Only 5·7 % of right colon cancers were in Stage A compared with 13·2 % in the left colon and 13·8 % in the rectum. In the right colon 31·2 % were in Stage $C_2$ or had palliative surgery compared with 22·3 % in the left colon and 17·8 % in the rectum. However, 32·4 % of rectal cases were in Stage $C_2$ in contrast to 16·9 % of left colon tumours and 20·5 % of right colon cases. Hence, although the proportion of advanced cases is highest in the right colon, an almost equal proportion of rectal cases had evidence of lymphatic spread. The proportion of cases with lymphatic spread was lowest in the left colon.

Thirdly, although there was a higher proportion of females than males with cancer in the right colon, it was found, as had been shown by Hughes (1966), that a higher proportion of these patients were over 75 years of age than were patients with tumours in other parts of the large bowel.

Cutler (1969) in a large statistical survey of cancers of the gut found a better overall survival in patients with colon tumours than with rectal ones. Muir (1956) reported similar results but Grinnell (1953) found little difference in survival between those with operable tumours in colon and rectum. Eker (1963) found that the prognosis was better in patients with right colon tumours than in those with tumours of either left colon or rectum, but numbers were fairly small. In a study of colon cancers Galante, Dunphy and Fletcher (1967) found survival to be highest after resection of tumours of the caecum and ascending colon.

Cancer of the colon is the second most common killing malignant disease in Britain accounting for some 14,000 deaths annually. Great strides have been made in treatment of this condition over the past 50 years and the improvement in resection rate and survival is striking. We have presented results from just over a 10-year period from a teaching hospital. The outcome would be much better if the number of patients presenting with advanced tumours could be reduced, but otherwise it is difficult to see more than minor improvements in survival occurring in the future.

We wish to thank the surgeons of the Middlesex Hospital who have generously agreed to our using their cases and our many colleagues in the Bland-Sutton Institute of Pathology, the Middlesex Hospital Medical School, who were involved in preparing and reporting bowel specimens. We wish to acknowledge help and encouragement from Dr. B. C. Morson and Dr. H. J. R. Bussey, and from Professor G. Dick in the preparation of this paper. Part of the expenses of this investigation were defrayed by the British Empire Cancer Campaign for Research.

### REFERENCES

BUSSEY, H. J. R., WALLACE, M. H. AND MORSON, B. C.—(1967) *Proc. R. Soc. Med.*, **60**, 208.
CUTLER, S. J.—(1969) *Surgery, St. Louis*, **65**, 740.
CUTLER, S. J. AND AXTELL, LILIAN M.—(1963) *J. Am. statist. Ass.*, **58**, 701.
DUKES, C. E.—(1940) *J. Path. Bact.*, **43**, 527.
DUKES, C. E. AND BUSSEY, H. J. R.—(1958) *Br. J. of Cancer*, **12**, 309.
EKER, R.—(1963) *Acta chir. scand.*, **126**, 636.

GALANTE, M., DUNPHY, J. E. AND FLETCHER, W. S.—(1967) *Ann. Surg.*, **165**, 732.

GRINNELL, R. S.—(1953) *Surgery Gynec. Obstet.*, **96**, 31.

HUGHES, E. S. R.—(1966) *Aust. N.Z. J. Surg.*, **35**, 3.

LILLEHEI, R. C. AND WANGENSTEEN, O. H.—(1955) *J. Am. med. Ass.*, **159**, 163.

MUIR, E. G.—(1956) *Br. med. J.*, ii, 742.

ROSENTHAL, L. AND BARONOFSKY, I. D.—(1960) *J. Am. med. Ass.*, **172**, 37.

WOLFMAN, E. F. J., ASTLER, V. B. AND COLLER, F. A.—(1957) *Surgery, St. Louis*, **42**, 846.